# Root Filling, Abscess, Etc.

**Published:** 1882-04

**Authors:** 


### ARTICLE VII.

### *Root-fillings, Abscess, Etc.*

---

If the nerve has been devitalized with arsenic, this may be removed in 24 or 48 hours after the application, but no attempt to remove the dead nerve should be made in less han a week. By this time the dead part has sloughed away from the live part at or beyond the apex of the root. A free, clear and as direct approach as possible to the nerve chamber and canal in the root should be made. One cannot expect good success if the approach or view is tortuous or obscure. Open well into the chamber with a cone-shaped bur—either a hand instrument, or a cone bur driven by the dental engine. When the entrance is well made, pass a nerve broach up to the edge of the fang. Let the barbs point towards the dentine and the smooth part of the broach against the nerve. In this way it will pass up to the end, in many cases, without pushing the dead nerve before it, and when the broach is twirled, several of the barbs will enter into and engage the dead nerve, and it will be drawn away entire. If it is not removed in this way, or if it is forced upwards, (or downwards, as the case may be,) before the broach, it will give pain in its removal, and the removal can only be effected piece by piece, with no certainty that all is removed, and also at the expense of much greater labor and time. When all *debris* is removed, wash out the canal with alcohol or chloroform. The latter has a very cleansing effect. In the single-rooted teeth—the six upper front teeth—the best locality for approach is through the palatial surface; and if the decay has reached the nerve through either proximate surface without involving much of the tissues of the teeth on these surfaces, and whether the nerve has died from exposure or from the application of a devitalizing agent, it would be best to fill the tooth with gold on these surfaces and then make an opening through the palatial surface, remove the dead nerve, as described above, and fill the tooth on this surface last. By these means a gutta-percha, or test filling may be inserted on the palatial surface, and the tooth left for a year or more with this test filling in; which may be readily and easily removed in case of any after-trouble. Of course, if the decay has involved much of the tissues of the tooth, it would be needless to weaken it further by making additional cavities, but best to remove the nerve through the cavity of decay. In the upper molar teeth the principal nerve to be removed is the one in the palatine root. The nerves in the two buccal roots are generally so fine and small that an entrance can be made into them with the finest nerve broach. Whenever they are large enough, however, they should be cleaned as thoroughly as any other root. I think the rubber dam should always be applied in all cases of the removal of dead or devitalized nerve, as it will assist materially in the operation and save much time. When the nerve has been destroyed with arsenic it is not necessary to go to the apex of the root; but all of the nerve in the root canal should be removed, except that hair-like part that occupies about the one-sixteenth part of an inch which is between the apex of the root and the end of the nerve canal.

In cases of abscess, where the fistula is external or shows a point of exit anywhere on the gum, creosote and iodine should be pumped freely through the root, until it makes its appearance in little bubbles or froth at the fistulus opening on the gum; but if this medicament refuses to pass through freely, the foramen at the end of the root should be enlarged or drilled through with a very fine small drill, and the medicine used until the pyogenic membrane at the end of the root, the lining membrane at the bottom of the alveolus, and the duct formed by the abscess, from the root to the gum, are all well soaked or cauterized with the caustic in the mixture above mentioned. The drilling through the root may be advised, if the root be straight, but if it be tortuous at the end, as upper laterals, incisors and cuspids sometimes are, it is doubtful if it is safe practice. One can generally tell, however, if the upper front teeth are tortuous at their apex, by the use of a very fine probe, which will readily pass through if they be straight, but which will stop if it be met by a crooked end.

Where there is no extended fistula, and all efforts at cure through the root prove unavailable, an external one may be made, by lancing the gum and drilling through until the drill reaches the abscess at the end of the root. This operation we consider dangerous in the lower bicuspid, as the inferior mental nerve artery and vein pass through the bone at about the locality, and the operation might be attended with considerable pain and hemorrhage if these vessels were cut.

The only painful part of this operation is the cutting through the gum. The drilling through the bone gives no pain. The *debris* should be washed away frequently, as the operation proceeds, with plain tepid water, or with a little warm salt and water. There are met with, occasionally, abscesses of such a thick, strong and leathery nature, that nothing can be done to cure them from the end of the root, where they hold with very great tenacity. These are to be regarded as hopeless cases, for it is with difficulty they are detached (even after extraction) from the end of the root with a sharp lancet; judge, then, the almost impossibility of doing this through the constricted opening, made by even a large-sized drill-hole. An abscess forms, sometimes in the molars, just behind the roots. When this is suspected, an opening to this part may be made with a drill through the nerve-chamber of the tooth, and the case treated from this location. The most unsatisfactory cases are the lower molars, which from the shape, of the roots, prevents as thorough manipulation as is desirable to effect a cure. Very little drilling or enlarging can be done on these roots, and their shape is such that all the *debris* of dead nerve is often impossible of removal. More dependence, in these cases, is to be placed on the cleansing effects of Labaraque's solution, creosote to disinfect, and alcohol to wash out, than on any actual removal of all portions, cut up into pieces with instruments.

Where the root is straight and single, as in the case of the six upper front teeth or the lower bicuspids and cuspids, they may be filled with wool, lead or gold, but cotton, well steeped in creosote, has proved, in many cases, as reliable a root filling as any that has been used. If the foramen at the end of the root is large, or has been enlarged by drilling through it, to cure abscess, wood or lead is preferable to cotton or gold. To fill the root with wood or lead, gauge the length of the root by passing a hook nerve extractor through it; draw it down until the hook stops; mark the length, and withdraw the instrument; dress down a piece of seasoned hickory with a file, and pass this into the root, and by means of the gauge, you can tell if the wood passes through the root or falls short of the apex.

When you have it fitted to the foramen pass your knife blade gently around about one-eighth of an inch from the end; dip this end in creosote and force it into place, give it a twirl and it will break off at the place where you knicked it with your knife, leaving the foramen well sealed and with nothing protruding beyond to irritate. Should you select lead to seal the apex with, it may be done in the same way as directed for wood; lead, however, may be drawn through a wire plate almost to the size needed and thus save much time in dressing it down to the proper size. One of the best instruments for enlarging a root canal is Palmer's nerve instrument No. ——. Talbot's reamers are very good, but the ends are so fine that they frequently break off in the operation of enlarging the canal.—*Dental Office and Laboratory.*

